# Manipulation of PK-M mutually exclusive alternative splicing by antisense oligonucleotides

**DOI:** 10.1098/rsob.120133

**Published:** 2012-10

**Authors:** Zhenxun Wang, Hyun Yong Jeon, Frank Rigo, C. Frank Bennett, Adrian R. Krainer

**Affiliations:** 1 Cold Spring Harbor Laboratory, Cold Spring Harbor, NY 11724, USA; 2 Watson School of Biological Sciences, Cold Spring Harbor, NY 11724, USA; 3 Graduate Program in Molecular and Cellular Biology, Stony Brook University, Stony Brook, NY 11794, USA; 4 Isis Pharmaceuticals, Carlsbad, CA 92008, USA

**Keywords:** alternative splicing, antisense oligonucleotides, cancer

## Abstract

Alternative splicing of the pyruvate kinase M gene involves a choice between mutually exclusive exons 9 and 10. Use of exon 10 to generate the M2 isoform is crucial for aerobic glycolysis (the Warburg effect) and tumour growth. We previously demonstrated that splicing enhancer elements that activate exon 10 are mainly found in exon 10 itself, and deleting or mutating these elements increases the inclusion of exon 9 in cancer cells. To systematically search for new enhancer elements in exon 10 and develop an effective pharmacological method to force a switch from PK-M2 to PK-M1, we carried out an antisense oligonucleotide (ASO) screen. We found potent ASOs that target a novel enhancer in exon 10 and strongly switch the splicing of endogenous PK-M transcripts to include exon 9. We further show that the ASO-mediated switch in alternative splicing leads to apoptosis in glioblastoma cell lines, and this is caused by the downregulation of PK-M2, and not by the upregulation of PK-M1. These data highlight the potential of ASO-mediated inhibition of PK-M2 splicing as therapy for cancer.

## 2. Introduction

Cancer cells preferentially use the glycolytic metabolic pathway with lactate generation, even under normal oxygen conditions [1]. This metabolic feature is termed the Warburg effect. Expression of the type II isoform of the pyruvate kinase M gene (*PKM2*, referred to here as *PK-M*) has been shown to mediate this effect, and to facilitate the proliferation of cancer cells *in vivo* [2].

The *PK-M* gene consists of 12 exons; exons 9 and 10 are alternatively spliced in a mutually exclusive fashion to give rise to M1 and M2 isoforms, respectively [3]. PK-M catalyses the final step in glycolysis to generate pyruvate and ATP from phosphoenolpyruvate and ADP [4]. Exons 9 and 10 each encode a 56-amino acid segment that confers distinctive properties to the respective PK-M isozymes. PK-M1 is constitutively active, whereas PK-M2 is allosterically regulated by fructose-1,6-bisphosphate levels and interaction with tyrosine-phosphorylated signalling proteins [5].

Consistent with the correlation between proliferation and PK-M2 expression, PK-M2 is highly expressed in embryonic tissues and in a broad range of cancer cells, whereas PK-M1 is predominantly expressed in terminally differentiated tissues [2,6]. In particular, the mammalian target of rapamycin pathway, which is a central mediator of cellular growth and proliferation, transcriptionally induces PK-M expression through the transcription factor hypoxia inducible factor-1α (HIF-1α) [7].

Paradoxically, downregulation of PK-M2 kinase activity is required for cancer cell growth. As PK-M2 is a rate-limiting enzyme in glycolysis, inhibition of PK-M activity decreases carbon flux through the catabolic glycolytic pathway, allowing upstream intermediates to be shunted to anabolic pathways, and thereby facilitating proliferation [2] and detoxification of reactive oxygen species (ROS) [8]. Apart from allosteric regulation, PK-M2 kinase activity can be suppressed in other ways, including growth-signalling-mediated inhibition through the binding of phosphorylated tyrosine proteins to its allosteric pocket [5], direct phosphorylation at Y105 [9], acetylation at K305 [10] and oxidation at C358 via ROS [8]. The multitude of avenues leading to PK-M2 inhibition underlies the importance of PK-M2 regulation and glycolysis in tumorigenesis.

PK-M2 has also been demonstrated to have critical functions beyond its kinase activity. In particular, PK-M2 can translocate into the nucleus and serve as a transcriptional co-activator for HIF-1α [11] and β-catenin [12] in mediating transactivation of targets important for tumour growth and proliferation. In spite of the importance of PK-M2 in tumorigenesis, little is known regarding alternative splicing of the PK-M pre-mRNA that predominantly generates the PK-M2 isoform in all cancer cells.

We and others previously showed that exon 9 inclusion, which generates the PK-M1 isoform, is actively repressed in cancer cells via the well-characterized PTB/nPTB and hnRNPA1/A2 splicing repressors [6,13]. We further showed that critical *cis*-elements that mediate the predominant PK-M2 splicing pattern in cancer cells are located in the exons, and we identified a potent exon-10 exonic splicing enhancer (ESE) that recruits the splicing activator SRSF3, promoting use of this exon [14].

Because of the importance of PK-M2 as a potential cancer drug target, and in order to systematically search for new splicing elements that mediate the PK-M2 dominant splicing patterns, we conducted an antisense oligonucleotide (ASO) screen targeting endogenous PK-M exon 10 to discover ASOs that switch the expression of the PK-M2 isoform to the PK-M1 isoform in cancer cells.

ASOs have generally been used to downregulate mRNA expression by homing to target mRNAs via Watson–Crick base pairing and inducing ribonuclease H (RNase H)-mediated degradation of the RNA [15]. Certain chemical modifications have been used to generate a class of nuclease-resistant ASOs with high affinity for their RNA targets and which do not operate through the RNase H mechanism. ASOs have been engineered with a wide range of chemical modifications, such as 2′-*O*-methyl (2′OMe), 2-*O*-methoxyethyl (MOE), peptide nucleic acid, locked nucleic acid and phosphorodiamidate morpholino. These ASOs work by direct sequence-specific annealing to the target transcript to block either splicing *cis*-elements, or ribosome recruitment so as to inhibit translation [16,17].

Our splicing ASO screen uncovered potent oligonucleotides that increase PK-M1 inclusion in cancer cell lines. We show that the effective ASOs principally target a new ESE in exon 10, and transfection of these ASOs into glioblastoma cell lines induces apoptosis. We demonstrate that this phenotype is caused by the ASO-mediated downregulation of PK-M2 expression, highlighting the therapeutic potential of the PK-M pre-mRNA as an ASO drug target.

## 3. Results

### 3.1. An exon 10-focused antisense oligonucleotide screen uncovers potent oligonucleotides that increase exon 9 inclusion and exon 10 skipping in cell lines

We previously reported that the critical *cis*-elements involved in the activation of exon 10 in proliferating cells are located within the exon [14]. To pinpoint the locations of these elements, we performed a systematic ASO walk along the entire length of PK-M exon 10. These ASOs have a phosphorothioate backbone and MOE at the 2′ ribose position, which allows them to bind to RNA targets with high affinity, while conferring resistance to both endogenous nucleases and to cleavage of the target RNA by RNase H [18,19]. The overlapping 15-mer ASOs were designed to cover the entire 167 nt exon 10 in 5 nt steps.

**Figure 1.**
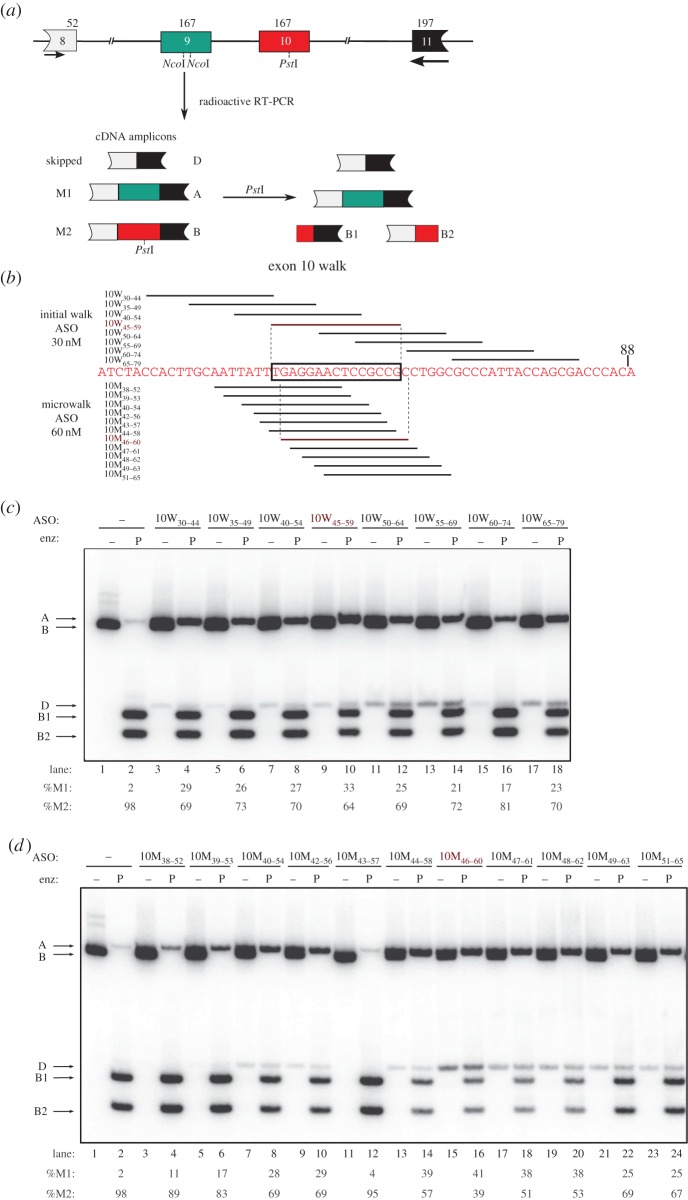
Antisense oligonucleotide (ASO) walk along the 10W region. (*a*) Diagram of the PK-M genomic region, and the RT-PCR assay to measure M1/M2 ratios. This region comprises introns 8, 9 and 10 (represented by the lines), intact exon 9 (green box), exon 10 (red box) and portions of exons 8 (white box) and 11 (black box). Numbers above the boxes show the length in nucleotides. Primers annealing to exons 8 and 11 used to amplify the endogenous PK-M transcript are represented by the arrows. cDNA amplicons generated after radioactive PCR are shown below and labelled accordingly. Three spliced species were observed: the shorter double-skipped species, comprising only exons 8 and 11 (D, 271 nt); M1, including exon 9 (A, 398 nt); and M2, including exon 10 (B, 398 nt). To distinguish between M1 and M2, a subsequent *Pst*I digest was carried out. Only M2 has a *Pst*I site, resulting in two cleavage products: B1 (213 nt) and B2 (185 nt), which are the 3′ and 5′ ends of M2, respectively. (*b*) Scheme of the ASO screen focused on the 10W region. The sequence of exon 10 from 25 to 88 nt upstream of the 5′ splice site (ss) is indicated in red. Stacked lines represent individual ASOs and are aligned to the complementary sequence in exon 10. The ASO names are indicated on the left, with the subscript numbers indicating the target-sequence coordinates. The initial ASO walk is represented at the top, with ASO 10W_45–59_ indicated in red and shown to be annealing to the 10W region (bounded by the rectangle) by vertical dashed lines. The microwalk ASOs are indicated below, and the complementary sequence targeted by ASO 10M_46–60_ is indicated with vertical dashed lines. (*c*,*d*) ASO walks. Radioactive RT-PCR and restriction digest of endogenous *PK-M* transcripts in HEK-293 cells after transfection of ASOs. Initial walk ASOs, transfected at 30 nM, are shown in (*c*), and microwalk ASOs transfected at 60 nm are shown in (*d*). RNAs were harvested from cells 48 h after transfection. The transfected ASO is indicated at the top. cDNA amplicons and fragments are indicated on the left. Lane numbers and quantifications are indicated at the bottom. Each product was quantified as a percentage of the total of M1, M2 and double-skipped species. %M1 and %M2 are shown. All standard deviations are ≤4% (*n* = 3).

To examine the effects of individual ASOs on endogenous PK-M transcripts, we transfected each ASO into HEK-293 cells at a nominal final concentration of 30 nM, and analysed the splicing of the endogenous PK-M transcripts by radioactive RT-PCR, 48 h after transfection (figure 1*a*). Some exon 10 ASOs strongly increased the proportion of PK-M1 mRNA, with a concurrent increase in the amount of double-skipped mRNA (joining of exons 8 and 11) and a decrease in PK-M2 mRNA, suggesting that these ASOs target functional ESEs in exon 10. The two most potent ASOs that decreased the proportion of PK-M2 mRNA were 10W_45–59_ (figure 1*c*, lane 10) and 10W_140–154_ (see the electronic supplementary material, figure S1*b*, lane 10). ASO 10W_140–154_ targets the previously characterized exon 10 SRSF3 motif [14], whereas ASO 10W_45–59_ targets a non-overlapping 15 nt region in the middle of exon 10 (termed the 10W region).

### 3.2. Antisense oligonucleotide microwalks centred on the 10W exonic splicing enhancer region

To find optimal ASOs that target the exon 10 region defined by ASO 10W_45–59_, we performed an ASO microwalk (figure 1*b*,*d*). Overlapping 15 nt ASOs targeting this region were designed in 1 nt steps. A total of 12 ASOs were synthesized for this region, and transfected into HEK-293 cells at a nominal concentration of 60 nM. ASO 10M_46–60_ was the most potent in increasing endogenous PK-M1 mRNA (figure 1*d*, lane 16) and decreasing PK-M2 mRNA abundance. We also performed a second microwalk centred on the region defined by ASO 10W_140–154_, and found that the ASO 10MS_139–153_ optimally abrogated the SRSF3-dependent ESE in exon 10 (see the electronic supplementary material, figure S1*c*, lane 10).

### 3.3. Antisense oligonucleotides 10W_45–59_ and 10M_46–60_ target a novel activation region of PK-M exon 10

**Figure 2.**
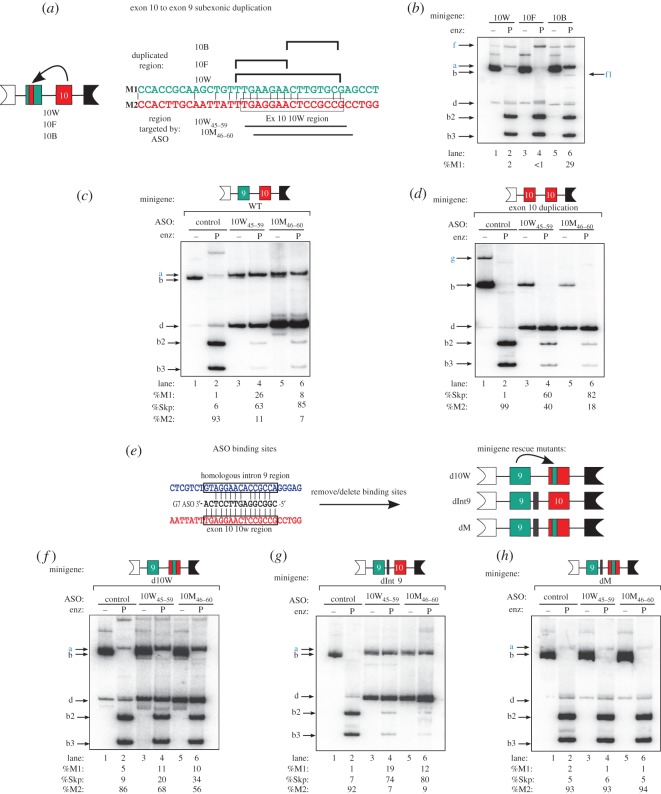
Characterizing the 10W ASO target region. (*a*) Scheme of method used to duplicate the exon 10 10W region into exon 9 in a minigene. The minigene comprises the same genomic region as indicated in figure 1*a*. To amplify minigene transcripts, we used a primer annealing to a vector-specific sequence upstream of the genomic insert, pcDNAF [14]. Minigene mutant names are indicated below. The indicated exon 9 (green) nucleotides at the top were mutated to the corresponding exon 10 (red) sequences on the right. The 10W minigene duplicates the entire exon 10 10W region into exon 9; the 10F minigene duplicates the first 8 nt of 10W45–59; and the 10B minigene duplicates the last 7 nt of 10W_45–59_. The ASOs that target 10W and the flanking regions are indicated below. (*b*) The 10W region is an exon 10 ESE. Mutant minigenes were analysed by transient transfection into HEK-293 cells, followed by radioactive RT-PCR and restriction digestion, as in figure 1. Constructs from (*a*) are labelled at the top. Labelled bands are indicated in lower case on the left and right, with important bands in blue font. %M1 is indicated at the bottom. Bands are as follows: uncut M1 fragment (**a**, 481 nt); uncut M2 fragment (**b**, 481 nt); *Pst*I-cleaved M2 5′ fragment (**b2**, 268 nt); *Pst*I-cleaved M2 3′ fragment (**b3**, 213 nt); a spliced mRNA that skips both exons 9 and 10 (**d**, 314 nt); an exon 9–exon 10 doubly included mRNA expressed from the 10B minigene (lanes 5 and 6) is indicated on the left (**f**, 648 nt). This band is sensitive to *Pst*I (**f1**, 435 nt). Standard deviations (s.d.) are 0.2%, 0.3% and 2.6% for 10G, 10F and 10B, respectively; *n* = 3. (*c*,*d*) Minigene transcript-level changes as a result of ASO co-transfection in HEK-293 cells. ASOs were transfected at a nominal final concentration of 60 nM. The wild-type (*c*) and exon 10 duplication (*d*) minigenes [14], together with the identity of the ASOs, are indicated at the top. Labelled bands are indicated in lower case on the left, with important bands in blue font. The exon 10–exon 10 doubly included mRNA in (*d*) expressed from the exon 10 duplication minigene is indicated on the right (**g**, 648 nt). %M1, %M2 or %Skp is indicated at the bottom. Standard deviations for (*c*) are 0.6%, 4.2% and 2.9% for control, 10W_45–59_ and 10M_46–60_, respectively; s.d. for (*d*) are 0.8%, 9.4% and 2.6% for control, 10W_45–59_ and 10M_46–60_, respectively; *n* = 3. (*e*) Diagram of a homologous potential cross-hybridizing region for ASOs 10W_45–59_ and 10M_46–60_ ASOs. Alignment of the sequences of ASO 10W_45–59_, the complementary region in exon 10 (indicated in red), and a homologous region in intron 9 (indicated in blue, 129–156 nt upstream of the exon 9 5′ss) is shown. Vertical lines indicate sequence identity. Diagram of minigene mutants used in (*f*–*h*) is shown on the left. d10W has the 10W ASO binding site in exon 10 removed and replaced by the corresponding region in exon 9. dInt9 has a 15 nt deletion of the homologous intron 9 region (*e*). dM has both mutations. (*f*–*h*) Minigene transcript-level changes as a result of ASO co-transfection in HEK-293 cells. ASOs were transfected at a final nominal concentration of 60 nM. The minigenes, together with the identity of ASOs, are indicated at the top. Labelled bands are indicated in lower case on the left, with important bands in blue font. ASOs and minigenes from (*e*) are indicated at the top, %M1 is indicated at the bottom and bands are indicated on the left. %M1, %M2 or %Skp is indicated at the bottom. Standard deviations for (*f*) are 1.2%, 1.6% and 1.5%; for (*g*) they are 0.4%, 2.0%, 3.9%; and for (*h*) they are 0.2%, 0.6% and 0.6%, corresponding to control, 10W_45–59_ and 10M_46–60_ ASOs, respectively; *n* = 3.

Because of the strong effect of ASOs 10W_45–59_ and 10M_46–60_ on PK-M splicing, we characterized the 15 nt 10W candidate ESE region in detail. To map the enhancer elements present in the 10W_45–59_ region, we took advantage of the high sequence identity between exons 9 and 10, and individually duplicated the entire 10W region of exon 10, or the first 7 nt (F7) or last 8 nt (B7) of the 10W region, into their corresponding exon 9 locations (figure 2*a*). Because of the low baseline PK-M1 inclusion from the wild-type minigene, any strong ESEs comprised by the candidate regions should lead to an increase in PK-M1 mRNAs expressed from the minigene. Duplication of the B7 region, but not the F7 and 10W region, led to increased exon 9 inclusion (figure 2*b*, compare lanes 2 and 4 with lane 6). This result suggests that the 8 nt B7 motif is a bona fide exon 10 ESE. Interestingly, duplication of F7 alone led to a further decrease in exon 9 inclusion, compared with the wild-type minigene (figure 1*c*, lanes 1 and 2), suggesting that F7 is an exon 10 splicing silencer that also represses the inclusion of exon 9 when placed in the context of the 10 W minigene.

### 3.4. Antisense oligonucleotides 10W_45–59_ and 10M_46–60_ interfere with exon 10 definition

To characterize the mechanism of action of ASOs 10W_45–59_ and 10M_46–60_ on the inclusion of exon 9 and skipping of exon 10, we co-transfected these ASOs with the previously characterized PK-M wild-type or duplicated exon 10 minigenes (figure 2*c*,*d*). The wild-type minigene comprises the flanking exons 8 and 11, and the complete genomic region between both exons, whereas the duplication construct has exon 10 cleanly replacing exon 9.

As expected, both ASOs 10W_45–59_ and 10M_46–60_ switched the splicing of the minigene transcript by simultaneously increasing the amount of M1 mRNA and decreasing the amount of M2 mRNA expressed from the wild-type minigene (figure 2*c*). However, ASO 10W_45–59_ increased exon 9 inclusion to a greater extent than 10M_46–60_, although the latter decreased exon 10 inclusion to a greater extent, resulting in higher levels of double-skipped (Skp) transcripts (figure 2*c*, compare lanes 4 and 6).

Co-transfection of ASOs 10W_45–59_ or 10M_46–60_ with the exon 10 duplication minigene interfered with the inclusion of exon 10, leading to a large increase in double-skipped species (figure 2*d*). ASO 10M_46–60_ was especially potent, nearly converting all the mRNA to the Skp isoform (figure 2*d*, compare lanes 4 and 6). These results suggest that both ASO 10W_45–59_ and ASO 10M_46–60_ interfere with the activation of exon 10.

### 3.5. The 10W region of exon 10 is the critical binding site for antisense oligonucleotides 10W_45–59_ and 10M_46–60_

Alignment of the 10W region with the PK-M exons 8–11 genomic region revealed a highly homologous region in intron 9 (figure 2*e*). To weigh the relative contributions of the exon 10 and intron 9 complementary regions for the effect of ASOs 10W_45–59_ and 10M_46–60_ on PK-M splicing, we made minigene mutations that eliminated the presumptive target sites in exon 10, intron 9 or both, and determined the effect of the ASOs on splicing of the mutant-minigene transcripts. Three mutants were generated (figure 2*e*): (i) we mutated the exon 10 10W region by duplicating the corresponding exon 9 region (termed the d10W construct); (ii) we introduced a 15 nt deletion in intron 9 that removed the homologous target region (termed the dInt9 construct); and (iii) we introduced both mutations in the same minigene (termed the dM construct).

There was a slight decrease in baseline minigene PK-M2 mRNA expressed from the d10W minigene (figure 2*f*, lane 2), suggesting that the duplicated exon 9 region contains weak repressor elements. This was not the case for the dInt9 construct, suggesting that this region alone does not have a major effect in dictating M1/M2 ratios.

The loss of the 10W binding site largely abrogated exon 9 inclusion and exon 10 skipping promoted by ASOs 10W_45–59_ and 10M_46–60_ (figure 2*f*, lanes 4 and 6). By contrast, removal of the intron 9 homologous region did not block the effect of ASOs 10W_45–59_ and 10M_46–60_ on splicing (figure 2*g*, lanes 4 and 6). However, when both binding sites were removed, the effect of ASOs 10W_45–59_ and 10M_46–60_ was completely abrogated (figure 2*h*, lanes 4 and 6). The above results indicate that ASOs 10W_45–59_ and 10M_46–60_ largely mediate exon 9 inclusion through the 10W complementary region in exon 10, though a small effect is attributable to cross-hybridization with the homologous region in intron 9.

### 3.6. Exon 10 antisense oligonucleotides switch PK-M RNA and protein expression in glioblastoma cells in a dose-dependent manner

**Figure 3.**
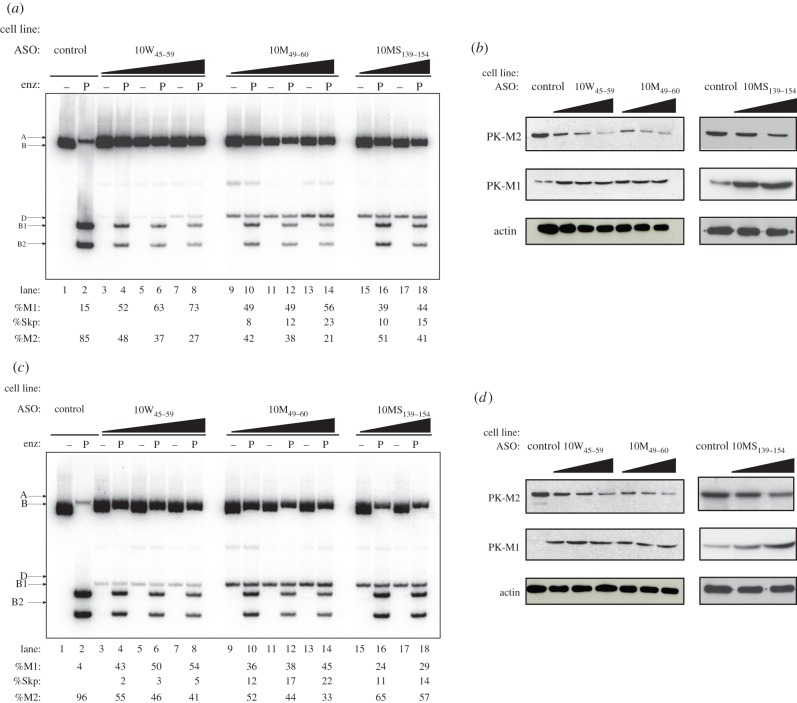
Effects of ASOs on PK-M mRNA and protein levels in glioblastoma cells. (*a*,*c*) Effects of ASOs 10M_46–60_, 10W_45–59_ and 10MS_139–153_ on endogenous PK-M mRNAs in (*a*) A172 and (*c*) U87-MG glioblastoma cells. Radioactive RT-PCR and restriction digest of endogenous *PK-M* transcripts were performed for the indicated cell lines 36 h after transfection of 30, 60 or 90 nM 10W_45–59_ or 10M_46–60_ ASO, or 60 or 90 nM 10MS_139–153_ ASO. The control ASO was transfected at 90 nM. %M1, %M2 and %Skp are indicated at the bottom. All s.d. are ≤4%; *n* = 3. (*b*,*d*) Immunoblot analysis of PK-M protein isoform levels in (*b*) A172 and (*d*) U87-MG cells transfected in (*a*,*c*). A representative blot from one of three independent experiments is shown. Antibodies used are indicated on the left.

To objectively compare the performance of ASOs targeting the 10W region versus those targeting the previously characterized SRSF3 region, we did side-by-side ASO transfections at different doses, with final concentrations of 30, 60 or 90 nM (figure 3*a*,*c*) in the glioblastoma cell lines A172 (figure 3*a*) and U87-MG (figure 3*c*). We chose to use glioblastoma cells because: (i) a characteristic splicing switch from PK-M1 to PK-M2 occurs during gliomagenesis [6,20]; and (ii) glioblastoma cell lines have a higher basal level of PK-M1 mRNA, which we expected to facilitate the ASO-mediated PK-M splicing switch [6].

As expected, there was a dose-dependent increase in exon 9 inclusion and exon 10 skipping in these cell lines, with ASOs 10W_45–59_ and 10M_46–60_ performing better than ASO 10MS_139–153_ (figure 3*a*,*c*, compare lanes 3–14 with lanes 15–18). Consistent with the minigene experiments, ASO 10W_45–59_ gave rise to more PK-M1 mRNA (figure 3*a*,*c*, lanes 4–8), whereas 10M_46–60_ gave rise to more double-skipped mRNA (figure 3*a*,*c*, lanes 9–14) and a larger decrease in PK-M2 mRNA. In addition, there was generally a larger switch in PK-M alternative splicing in these cells, compared with HEK-293 cells.

Using isoform-specific antibodies, we measured the amount of PK-M1 and PK-M2 proteins in the same lysates as above (figure 3*b,d*). As expected, PK-M1 and PK-M2 protein isoform levels closely mirrored their mRNA levels. There was detectable PK-M1 protein after transfection of each of the three ASOs, but ASO 10M_46–60_ resulted in the greatest decrease in PK-M2 levels. Downregulation of PK-M2 protein was also observed by indirect immunofluorescence staining with the PK-M2 antibody when either ASO 10W_45–59_ or 10M_46–60_, but not the control ASO, was transfected into A172 or U87-MG cell lines (see the electronic supplementary material, figure S2).

### 3.7. Antisense oligonucleotide treatment induces apoptosis in glioblastoma cells

**Figure 4.**
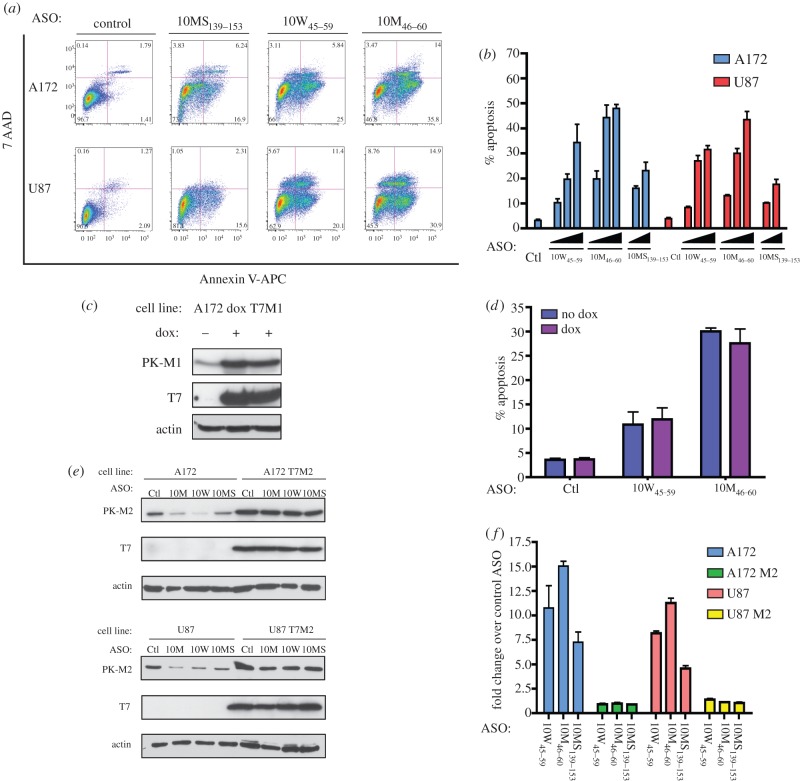
Effects of ASOs on glioblastoma cells. (*a*,*b*) Exon 10 ASOs induce apoptosis in glioblastoma cells. (*a*) U87-MG or A172 cells were transfected with the indicated ASOs at a nominal final concentration of 90 nM, stained with Annexin V-APC/7-AAD 36 h after transfection and analysed by flow cytometry. The percentage of Annexin-V-positive cells is indicated for the two right quadrants in each plot. Each plot is a representative of three biological replicates. (*b*) ASO-induced apoptosis is dose-dependent. The indicated cells were transfected with ASOs 10W_45–59_ or 10M_46–60_ at 30, 60, or 90 nM, or ASO 10MS_139–153_ at 60 or 90 nM. The control (Ctl) ASO was transfected at 90 nM. The percentage of Annexin-V-positive cells is plotted for each condition. The identity and dose of ASOs are indicated below the *x*-axis. Error bars represent s.d. (*n* = 3). (*c*,*d*) Role of PK-M1 protein isoform in apoptosis induction. (*c*) Immunoblot analysis of A172 cells stably transduced with rtTA and doxycycline (dox)-inducible human T7-tagged PK-M1 cDNA. Cells were grown in parallel, with or without doxycycline, and harvested after 72 h. Antibodies used are indicated on the left. (*d*) Cells were grown as in (*c*) and then transfected with the indicated ASOs at 60 nM. Cells were then stained and analysed for Annexin V 36 h after transfection. Histograms indicate the percentage of Annexin-V-positive cells for each condition. Doxcycyline on/off conditions are indicated on the left. Error bars represent s.d. (*n* = 3). (*e*,*f*) Role of PK-M2 protein isoform in apoptosis induction. A172 or U87 cells were stably transduced with T7-tagged human PK-M2 cDNA; transductants and the parental cell lines were transfected with the indicated ASOs at a nominal final concentration of 90 nM. (*e*) Immunoblot analysis of cells transfected with the indicated ASOs. Antibodies used are indicated on the left. (*f*) ASO-transfected cells were analysed for Annexin V as in (*d*). Histograms indicate the fold increase in Annexin-V-positive cells, compared with control ASO, for each cell line. Error bars represent s.d. (*n* = 3).

Upon transfection of ASO 10M_46–60_, but not the control ASO, into A172 cells, cleaved PARP appeared as early as 24 h post-transfection (see the electronic supplementary material, figure S3*a*), indicating that the cells were undergoing apoptosis. To confirm and quantitate this observation, we performed Annexin V staining assays of A172 and U87-MG cells transfected with ASO 10W_45–59_, 10M_46–60_ or 10MS_139–153_, 36 h after transfection (figure 4*a*). As expected, the proportion of Annexin-V-positive cells increased in an ASO dose-dependent manner, indicating that ASO-mediated switching of PK-M splicing induces apoptosis in these cell lines. ASO 10M_46–60_ was the most potent in inducing apoptosis among the three ASOs tested.

### 3.8. Antisense oligonucleotide-mediated apoptosis in glioblastoma cells is caused by the downregulation of endogenous PK-M2 expression

ASO transfection elicited a simultaneous increase in PK-M1 expression and a decrease in PK-M2 expression. To pinpoint which of these changes is responsible for inducing apoptosis in glioblastoma cells, we generated stable cell lines that express human PK-M1 cDNA in a doxycycline-inducible manner (figure 4*c*), or PK-M2 cDNA constitutively (figure 4*e*). To investigate the role of PK-M1 in ASO-mediated apoptosis, we added doxycyline to the PK-M1 inducible cells for three days, and then treated them with ASO 10W_45–59_, 10M_46–60_ or control ASO. There was a similar increase in the number of Annexin-V-positive cells in the cells that did or did not overexpress PK-M1, suggesting that PK-M1 induction did not cause apoptosis in these cells (figure 4*d*).

To investigate the role of PK-M2 downregulation in apoptosis, we overexpressed PK-M2 in U87-MG and A172 cells, and then treated them with the maximum dose of ASO 10W_45–59_, 10M_46–60_ or 10MS_139–153_ (figure 4*e*). Overexpression of PK-M2 in both cell lines rescued the cells from ASO-mediated apoptosis, leading to a decrease in the number of Annexin-V-positive cells to baseline levels (figure 4*f*). To confirm this result, we knocked down PK-M2 by siRNA in A172 cells. Knockdown of PK-M2 also led the appearance of cleaved PARP after 48 h (see the electronic supplementary material, figure S3*b*). These observations indicate that the downregulation of PK-M2 expression, but not the induction of PK-M1 expression, leads to apoptosis in these cell lines.

## 4. Discussion

We performed a systematic exon 10 ASO screen, and developed potent ASOs that increase the inclusion of exon 9 and skipping of exon 10 in transfected cell lines. We found that these ASOs switch PK-M splicing especially effectively in glioblastoma cells, and they induce apoptosis. Finally, we showed that it is the downregulation of PK-M2 expression that causes this apoptosis.

Glioblastomas exhibit a high rate of glycolysis, even under normal oxygen conditions [6]. Consistent with this phenomenon, the splicing profile of glioblastoma cell lines, as with other cancer cell lines, is predominantly PK-M2. Because PK-M2 is a rate-limiting glycolytic enzyme, targeting this isoform might inhibit the entire glycolytic pathway, leading to apoptosis, as previously observed by glucose withdrawal in glioblastoma cells *in vitro* [21]*.* The replacement of PK-M2 in tumour cells with PK-M1 also leads to a shunting of glycolytic end-products into the mitochondria [5], phenocopying the apoptotic phenotype displayed by glioblastoma cells after treatment with dichloroacetic acid [22,23]. In addition, because PK-M2 also functions as a co-activator of HIF-1 [11] and β-catenin [12] transactivation, reducing the level of PK-M2, as opposed to just inhibiting its kinase function, might also interfere with anti-apoptotic and pro-proliferative functions [24] mediated by these factors.

How does blocking the activation of the PK-M exon 10 10W region result in activation of exon 9? This may be explained in terms of 3′ splice site (ss) competition, such that the loss of exon 10 definition leads to more efficient recognition of the exon 9 3′ss by the spliceosome. This was previously observed upon knockdown of SRSF3 [14]—a recently discovered exon 10 activator—leading to a large increase in PK-M1 mRNA levels in HEK-293 cells. Although here we have successfully targeted a novel exon 10 ESE motif, we do not presently know which splicing factor(s) actually binds to this 10W ESE. In addition, even though the highly homologous 10W-like region in intron 9 does not by itself play a strong role in exon 10 activation and/or exon 9 repression, rescue experiments indicate that it works in tandem with the 10W ESE in exon 10. It will be of interest to identify the factors responsible for recognition of the exon 10 10W ESE and of the related element in intron 9, so as to elucidate the exact mechanism of exon 10 activation mediated by these elements. However, due to the complicated mechanism controlling mutually exclusive exon use in this gene, we cannot rule out the potential influence of RNA secondary and/or tertiary structures in mediating exon 10 activation. Consequently, apart from or in conjunction with disrupting RNA–protein interactions, these ASOs might also work by disrupting putative RNA higher-order structure present in exon 10.

Additionally, the 10W region appears to comprise adjacent silencer (10F) and enhancer (10B) regions. Although it is clear from the minigene-duplication experiments that the silencing effect of 10F is masked by 10B in the native context of exon 10, but is dominant in the context of exon 9, the mechanism responsible for this context-specific repression remains unknown.

Although certain exon 10 ASOs increase the expression of PK-M1 protein isoform and decrease that of PK-M2 protein isoform, we cannot rule out that these ASOs might partially disrupt PK-M2 mRNA export and translation by remaining hybridized to the spliced mRNA. Although previous experiments targeting *SMN2* pre-mRNA argued against the influence of analogous ASOs on mRNA transport and translation [25], further work will be necessary to establish whether the PK-M ASOs act solely at the level of modulating alternative splicing.

Although siRNA-mediated knockdown of PK-M2 in the NCI 60 panel of cell lines has also been reported to induce apoptosis [26], that strategy might be less effective, considering that the onset of apoptosis was greatly delayed (144 h versus 36 h in the present study). In addition, because glioblastoma cells can freely take up certain chemically modified ASOs *in vivo* [27] without the aid of delivery agents, ASO-mediated PK-M switching could potentially be a particularly effective therapeutic strategy.

Although the ASOs we have screened are potent enough to switch the PK-M splicing profile of glioblastoma cells from PK-M1 to PK-M2 *in vitro*, the effects of these ASOs on cells and tissues that predominantly express PK-M1 are presently unknown. This will be difficult to test *in vitro*, because all cell lines assayed to date predominantly include exon 10 and express the PK-M2 isoform, regardless of their transformation status [26] (data not shown). However, given our successful experience with intracerebroventricular administration of ASOs in rodents and non-human primates [28–31], it should be possible in the future to assess the safety of PK-M2 reduction in normal CNS tissues, as well as to evaluate the efficacy of these ASOs in mouse glioblastoma models.

## 5. Experimental procedures

### 5.1. Cells and transfections

HEK-293, U87-MG and A172 cells were obtained from ATCC and grown in DMEM supplemented with 10 per cent (v/v) FBS, penicillin and streptomycin, at 37°C and 5 per cent CO_2_. 5 μg of minigene plasmid per 10 cm dish, or 1 μg per 6-cm dish, was transiently transfected using Lipofectamine 2000 (Invitrogen). Total RNA from transfected cells was harvested after 48 h. For ASO transfections, 2 × 10^6^ cells were first plated in 6 cm dishes, and then transfections were performed using an ASO : Lipofectamine 2000 ratio of 20 pmoles : 1 μl. For Annexin V assays and RT-PCR experiments in glioblastoma cells, total RNA and protein from transfected cells were harvested after 36 h.

### 5.2. Oligonucleotide synthesis

2′-*O*-methoxyethyl-modified oligonucleotides with a phosphorothioate backbone were synthesized on an Applied Biosystems 380B automated DNA synthesizer (Applied Biosystems, Foster City, CA) and purified as described [32]. The oligonucleotides were dissolved in water. The sequence of the control ASO is 5′-TCATTTGCTTCATACAGG-3′.

### 5.3. RNA interference

Four siRNAs targeting exon 10 of human *PKM2* were obtained from Sigma Genosys, and have the sense-strand sequences 5′-CCAUAAUCGUCCGCACCAA-3′ (M2si1), 5′-CAUCUACCACUUGCAAUUA-3′ (M2si2), 5′-CCGUGGAGGCCUCCUUCAA-3′ (M2si3) and 5′CUUGCAAUUAUUUGAGGAA-3′ (M2si4). 4 × 10^6^ A172 cells in six-well plates were transfected with 400 pmol of siRNA duplex using Lipofectamine 2000. Cells were harvested 48 h later.

### 5.4. Retrovirus transduction

To generate cell lines that overexpress human PK-M1 isoform in a doxycyline-dependent manner, A172 cells were first infected with MSCV-rtTA-hygro virus, and selected in hygromycin for two weeks. Human PK-M1 cDNA was amplified from A172 cells transfected with 10M_46–60_ ASO using the following primer pair: hPKT7cDNAF (5′-GGGGAACTCGAGATGGCTTCTAGGATGGCATCGATGACAGGTGGCCAACAGATGGGCATGTCGAAGCCCCATAGTGAAGCCG-3′) and hPKT7cDNAR (5′-GGGGAAGAATTCTCACGGCACAGGAACAACACGCATG-3′), with Phusion high-fidelity DNA polymerase (Finnzymes). The resulting amplicon containing the T7 tag was gel-purified and cloned between the *Eco*RI and *Xho*I sites of the retroviral TtiGP plasmid. A172-rTA cells were then infected with TtiGP-PKM1 virus. To make cell lines constitutively overexpressing human PK-M2, PK-M2 cDNA from A172 cells was amplified using the same primers and cloned between the *Eco*RI and *Xho*I sites of the retroviral PIG plasmid. A172 and U87-MG cells were then infected with the PIG-PKM2 virus. All infected cells were then selected with 100 μg ml^−1^ puromycin for 3 days. All plasmids were sequenced to confirm their identities.

### 5.5. Immunoblotting

Cells were lysed in SDS, and total protein concentration was measured by the Bradford assay. 5–30 μg of total protein was separated by SDS–PAGE and transferred onto nitrocellulose, followed by blocking with 5 per cent (w/v) milk in Tris-buffered saline with Tween-20, probing with the indicated antibodies, and visualization by enhanced chemiluminescence (Roche). Primary antibodies were: β-actin (Genscript, mAb, 1 : 10 000); PK-M2 (Cell Signaling Technology, rAb, 1 : 2000); PARP (Cell Signaling Technology, rAb, 1 : 1000); T7 (mAb, 1 : 1000) and PK-M1 (ProteinTech, rAb, 1 : 1000). Secondary antibodies were goat anti-mouse or anti-rabbit HRP conjugates, 1 : 20 000 (Bio-Rad).

### 5.6. Minigene construction

DNA oligonucleotides were obtained from Sigma Genosys. The *PK-M2* minigene was constructed by amplifying a 6.4 kb *PK-M* exon 8–11 fragment from human genomic DNA (Promega) using Phusion high-fidelity DNA polymerase and primers PKMinigeneF (5′-GGGGAAAGATCTGCCACCATGGGAGAAACAGCCAAAGGGGAC-3′) and PKMinigeneR (5′-GGGGAACTCGAGCTAGACATTCATGGCAAAGTTCACC-3′). The product was then digested and cloned between the *Bam*HI and *Xho*I sites of pcDNA3.1+ (Invitrogen). For exon-duplication and intron-deletion constructs, the upstream KpnI site 1552 nt downstream of exon 8 was removed by a 1 nt deletion, and an *Eco*RV restriction site was generated 90 nt upstream of exon 9 by a 2 nt insertion to create a modified wild-type minigene. To generate the 10W, 10B7 and 1010F7 constructs, modified exon 9 fragments were generated by annealing the following oligonucleotides: 10W F (5′-CCCTAAACCTTACAGATAGCTCGTGAGGCTGAGGCAGCCATGTTCCACCGCAAGCTGTTTGAGGAACTCCGCCGAGCCTCAAGTCACTCCACAGACCTCATGGAAGCCAT-3′), 10F7F (5′-CCCTAAACCTTACAGATAGCTCGTGAGGCTGAGGCAGCCATGTTCCACCGCAAGCTGTTTGAGGAACTTGTGCGAGCCTCAAGTCACTCCACAGACCTCATGGAAGCCAT-3′), 10B7F (5′-CCCTAAACCTTACAGATAGCTCGTGAGGCTGAGGCAGCCATGTTCCACCGCAAGCTGTTTGAAGAACTCCGCCGAGCCTCAAGTCACTCCACAGACCTCATGGAAGCCAT-3′), with Exon 9Rev oligo (5′-CCCTTAGGGCCCTACCTGCCAGACTCCGTCAGAACTATCAAAGCTGCTGCTAAACACTTATAAGAAGCCTCCACGCTGCCCATGGCCATGGCTTCCATGAGGTCTG -3′), and amplifying using Ex10ADupF (5′-TTCCCCATTCTGTCTTCCCATGTGTTGTGTCTCGTTTTTTTCCTCCTCCTTCCCTCTTCCTTGCCCCCTCTTCCCCTAAACCTTACAG-3′) and Ex10ADupR (5′-AGTGTTACCTGCCCTT AGGGCCCTAC-3′). The 106 nt oligonucleotide carries mutations that duplicate specific stretches of exon 10 over the corresponding region of exon 9. Another fragment was amplified from the wild-type minigene using the following primer pairs: Ex10BF 5′-GTAGGGCCCTAAGGGCAGGTAACAC-3′ and RKpnI (5′-GGGGAAGGTACCACTGAGCAGGGCATT-3′). Both fragments were then gel-purified, and subjected to a second overlap-extension (OE) PCR using the end primers FEcoRV (5′-GGGGAAGATATCAATTCCCCATTCTGTCTTCCCATGT-3′) and RKpnI (5′-GGGGAGGTACCACTGAGCAGGGCATT-3′).

To generate the d10W minigene construct, a modified exon 10 fragment was constructed by annealing d10W F (5′-ATGTTGCTCCCCTAGATTGCCCGTGAGGCAGAGGCTGCCATCTACCACTTGCAATTATTTGAAGAACTTGTGCGCCTGGCGCCCATTACCAGCGACCCCACAGAAGCCAC-3′) with Exon 10 Rev (5′-CGCTGCCGCCTCCTACCTGCCAGACTTGGTGAGGACGATTATGGCCCCACTGCAGCACTTGAAGGAGGCCTCCACGGCACCCACGGCGGTGGCTTCTGTGGGGTCGCT-3) and amplifying using Ex9ADupF (5′-TGGACGGATGTTGCTCCCCTAG-3′) and Ex9ADupR (5′-GGT ACCACTGAGCAGGGCATTCCAGGGAGCCGCTGCCGCC TCCTAC-3′). The 108 nt oligonucleotide carries mutations that duplicate specific stretches of exon 9 over the corresponding region in exon 10. Another fragment was amplified from the wild-type minigene using the following primer pairs: FEcoRV and Ex9BR (5′-GTAGGGCCCTAAGGGCAGGTAACAC-3′). Both fragments were then gel-purified and subjected to a second OE PCR using the FEcoRV and RKpnI primers.

To generate the dInt9 mutant, two fragments were generated from the wild-type minigene construct, using the following primer pairs: FEcoRV and PKMdelB12R (5′-TGCCCTGCCATGACCTCCCAGACGAGAAGAGGCTCTGTGCCCAG-3′) and PKMdelB125 (5′-ACAGAGCCTCTTCTCGTCTGGGAGGTCATGGCAGGGCAG-3′). To generate the dM double mutant, the same two fragments were generated from the d10W minigene. Both fragments were then gel-purified and subjected to a second OE PCR using FEcoRV and RKpnI. All generated fragments were then cloned between the *Eco*RV and *Kpn*I sites of the modified wild-type minigene plasmid.

### 5.7. RT-PCR

Total RNA (2 μg) was extracted from cell lines using Trizol (Invitrogen). Contaminating DNA was removed with DNAase I (Promega). Reverse transcription was carried out using ImPromp-II reverse transcriptase (Promega). Semi-quantitative PCR using Amplitaq polymerase (Applied Biosystems) was performed by including [α-^32^P]-dCTP in the reactions. The human-specific primer sets used to amplify endogenous transcripts anneal to *PK-M* exons 8 and 11, and their sequences are: hPKMF, 5′-AGAAACAGCCAAAGGGGACT-3′; hPKMR, 5′-CATTCATGGCAAAGTTCACC-3′. To amplify minigene-specific transcripts, the forward primer was replaced with a primer annealing to the pcDNA3.1(+) vector, pcDNAF (5′-TAATACGACTCACTATAGGG-3′). After 28 amplification cycles for minigene-derived transcripts, and 27 cycles for endogenous transcripts, the reactions were divided into two aliquots for digestion with *Pst*I (New England Biolabs) or no digestion. The products were analysed on a 5 per cent native polyacrylamide gel, visualized by autoradiography and quantified on a Typhoon 9410 phosphorimager (GE Healthcare) using Multi Gauge software v. 2.3. The percentage M1 mRNA in endogenous transcripts was calculated using the GC-content-normalized intensities of the top undigested band (M1, A), the bottom two digested bands (M2, B1 B2) in the *Pst*I-digest lanes and the double-skipped species (D), if detectable. The percentage M1 mRNA from minigene transcripts was calculated using the GC-content-normalized intensities of the top undigested band (**a,** M1) and other higher-mobility digested bands corresponding to M2 and its variant species (**b–g**, as described above) in the *Pst*I-digest lanes. All the PCR products were gel-purified, cloned and sequenced to verify their identities.

### 5.8. Annexin V Assays

For Annexin V/7-AAD assays, cells were stained with Annexin V-APC and 7-AAD, and analysed for apoptosis by flow cytometry using an LSRII Cell Analyzer (Becton–Dickinson). Briefly, 10^6^ cells were collected 36 h after transfection, washed twice with phosphate-buffered saline (PBS), resuspended in 1 × Binding Buffer (10 mM HEPES, pH 7.4, 140 mM NaCl, 2.5 mM CaCl_2_) and incubated with 5 μl each of Annexin V-APC antibody and 7-AAD (Becton–Dickinson) in the dark for 15 min. Both early-apoptotic (7AAD-/Annexin V+) and late-apoptotic (7AAD+/Annexin V+) cells were included in the quantification.

### 5.9. Immunofluorescence

Cells were first transfected with ASOs as above, and then plated onto four-well culture slides (BD Biosciences) 24 h post-transfection. At 36 h post-transfection, cells were washed with PBS and fixed with 3.7 per cent formaldehyde in PBS for 20 min. Cells were then permeabilized in 0.1 per cent Triton X-100 in PBS for 10 min after washing in PBS, blocked for 20 min in blocking buffer (1% goat serum in PBS) and then incubated overnight with rabbit monoclonal anti-PKM2 antibody (Cell Signaling Technology). After washing three times with PBS, the cells were then incubated for 1 h in blocking buffer containing Alexa Fluor 594 conjugated goat anti-rabbit secondary antibody (Molecular Probes/Invitrogen). Cells were washed with PBS, and then culture slides were disassembled and mounted with Prolong Gold mounting solution containing DAPI (Molecular Probes/Invitrogen). Cells were analysed using a Zeiss Axioplan1 upright fluorescent microscope.

## 6. Acknowledgements

We thank members of the Krainer laboratory, in particular Yimin Hua and Kentaro Sahashi, for helpful discussions. A.R.K. was supported by NCI grant no. CA13106 and by the St Giles Foundation. Z.W. is supported by a National Science Scholarship from the Agency for Science, Technology and Research, Singapore.

## Supplementary Material

Supplementary Figure 1

## Supplementary Material

Supplementary Figure 2

## Supplementary Material

Supplementary Figure 3
