# Supplementary figures and images for: Manipulation of PK-M mutually exclusive alternative splicing by antisense oligonucleotides
Source: Open Biol. 2012 Oct;2(10):120133. doi: 10.1098/rsob.120133 (PMC3498831; doi:10.1098/rsob.120133)

Supplementary Figure 1

a

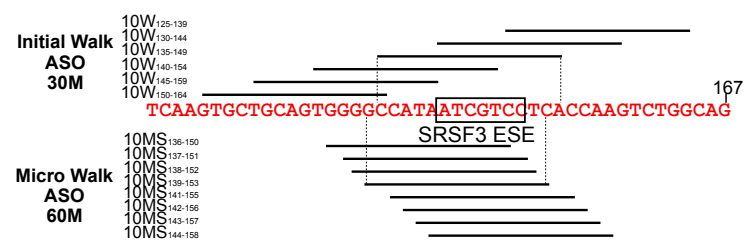

b

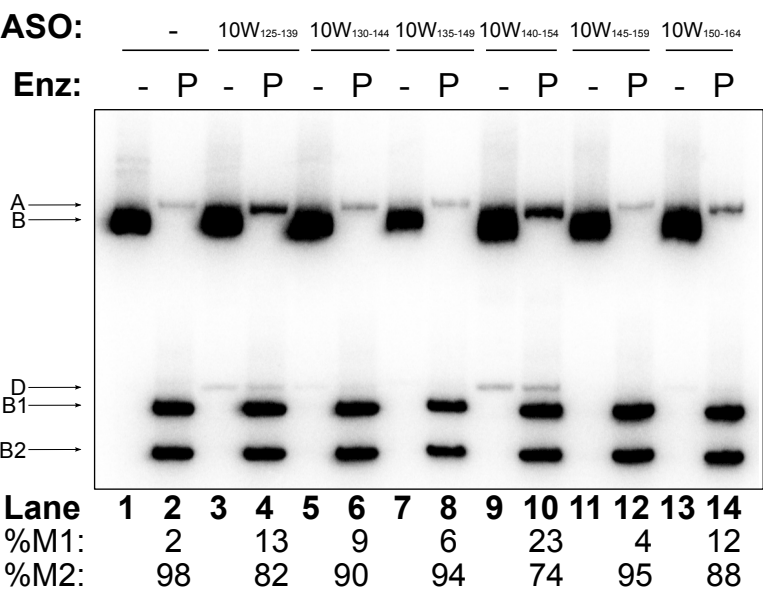

c

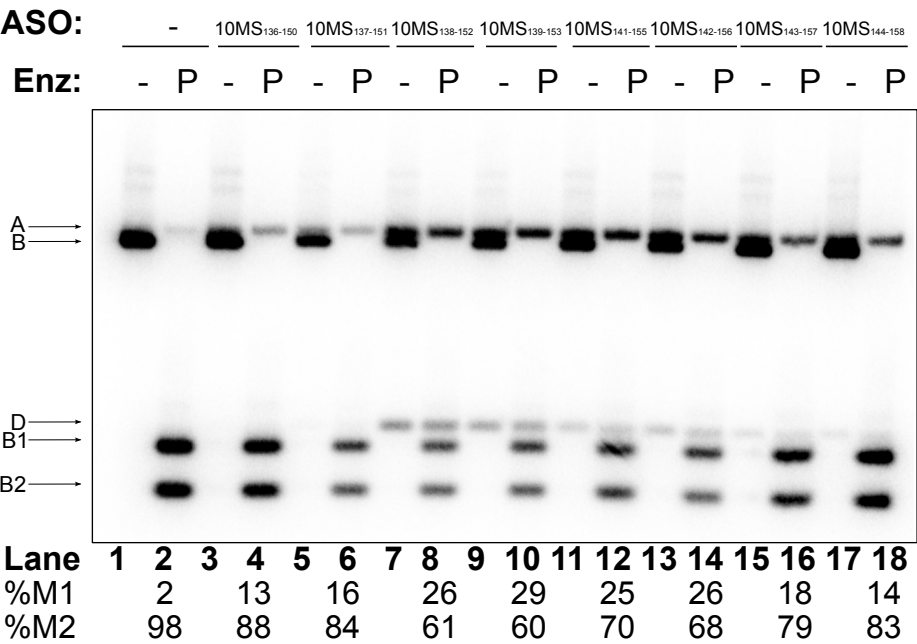

Supplement: Supplementary Figure 1 [file rsob120133-s1.pdf]

Supplementary Figure 2

a

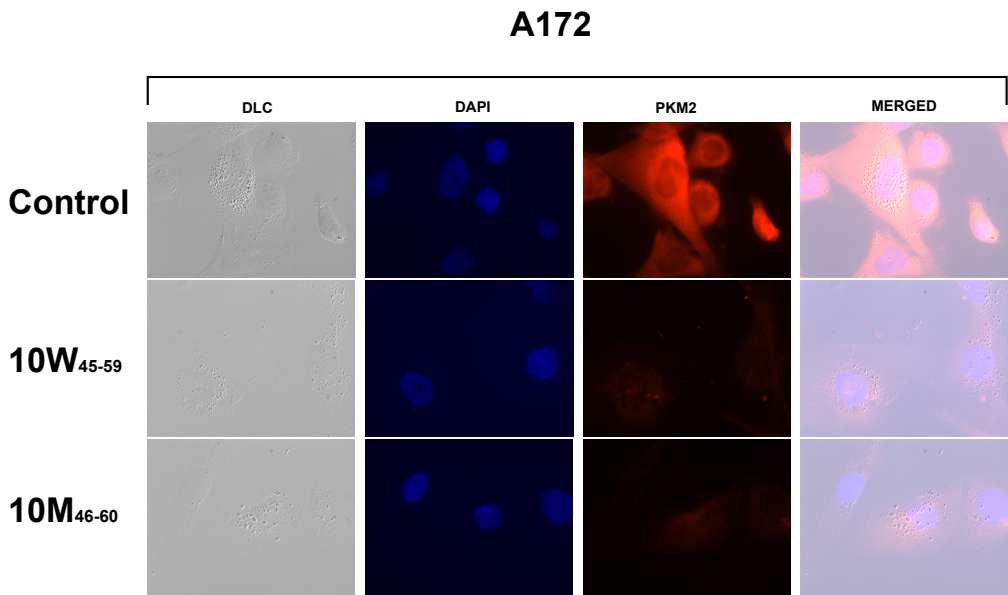

b

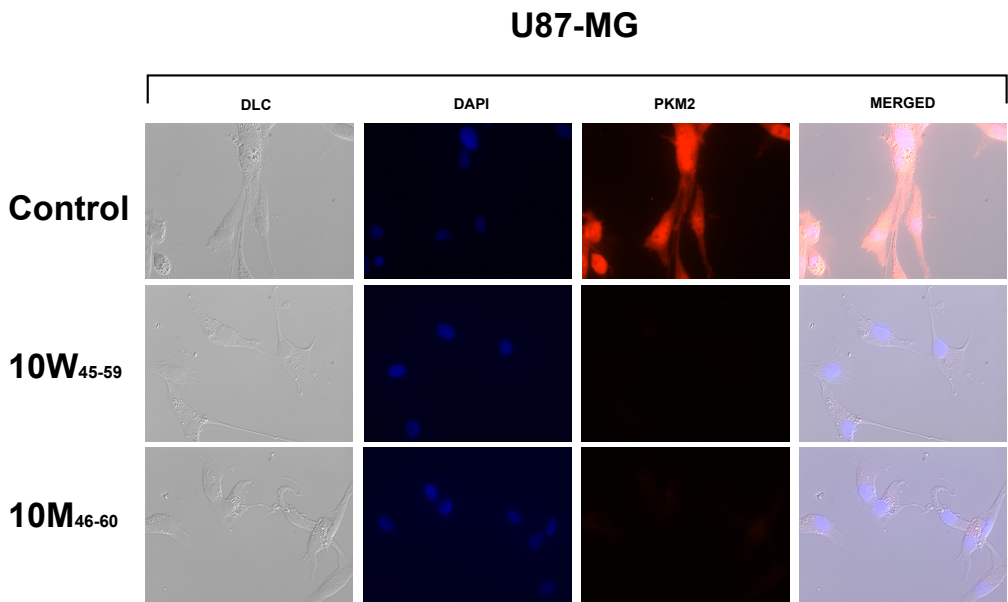

Supplement: Supplementary Figure 2 [file rsob120133-s2.pdf]

## Supplementary Fig 3

**a**

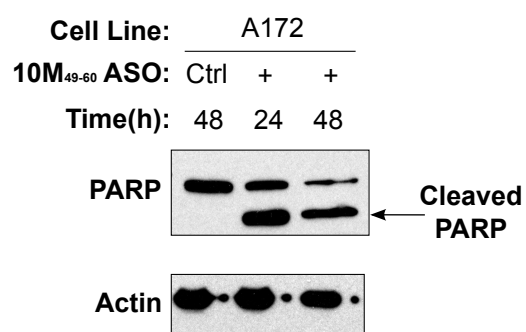

**b**

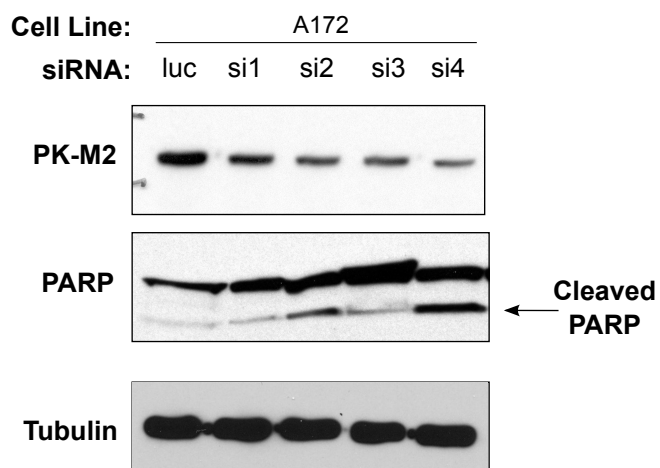

Supplement: Supplementary Figure 3 [file rsob120133-s3.pdf]
